# Association between Parkinson’s Disease Medication and the Risk of Lower Urinary Tract Infection (LUTI): A Retrospective Cohort Study

**DOI:** 10.3390/jcm11237077

**Published:** 2022-11-29

**Authors:** Niklas Gremke, Sebastian Griewing, Marcel Printz, Karel Kostev, Uwe Wagner, Matthias Kalder

**Affiliations:** 1 Department of Gynecology and Obstetrics, University Hospital Marburg, Philipps-University Marburg, Baldingerstraße, 35043 Marburg, Germany; 2 Department of Neurology, University Hospital Marburg, Philipps-University Marburg, Baldingerstraße, 35043 Marburg, Germany; 3 Epidemiology, IQVIA Commercial GmbH & Co. OHG, Main Airport Center, Unterschweinstiege 2-14, 60549 Frankfurt am Main, Germany

**Keywords:** Parkinson’s disease (PD), levodopa-based therapy, autonomic dysfunctions, lower urinary tract infections (LUTI), antibiotic drug prescriptions

## Abstract

Background: The occurrence of autonomic dysfunctions (e.g., urological dysfunctions) is a common phenomenon during the course of Parkinson’s disease (PD) and resulting complications such as lower urinary tract infections (LUTI) are one of the leading causes of hospitalizations and mortality in patients with the condition. Therefore, the aim of this retrospective cohort study was to compare the most common levodopa-based treatment regimens (DOPA decarboxylase inhibitor (DCI) + carbidopa or benserazide) and to analyze the incidence of LUTI and antibiotic prescriptions in patients receiving the respective treatments. Methods: This study was based on data from the Disease Analyzer database (IQVIA) and included adult patients (≥18 years) with an initial prescription of levodopa therapy including fixed-dose levodopa/DCI combinations in 1284 general practices in Germany between January 2010 and December 2020. Conditional Cox regression models were used to analyze the association between levodopa/DCI combinations and LUTI incidence and antibiotic prescriptions. Results: Compared to levodopa + carbidopa, levodopa + benserazide therapy was significantly and negatively associated with LUTI (HR: 0.82; 95% CI: 0.71–0.95). This association was stronger in women (HR: 0.77; 95% CI: 0.65–0.92) than in men (HR: 0.93, not significant). Conclusions: Especially in women, receiving levodopa + benserazide prescriptions was associated with a lower LUTI incidence. It is important for clinicians to keep this in mind, since LUTI is a leading cause of hospitalizations, morbidity, and mortality in patients with PD.

## 1. Introduction

Parkinson’s disease (PD) is a neurodegenerative disorder that has had a fast-growing incidence in recent decades. The number of patients with PD doubled to more than 6 million between 1990 and 2015, making PD the fastest-growing neurological disorder worldwide [1,2]. The pathophysiology of PD is characterized by a neuronal α-synuclein misfolding and/or aggregation (key component of Lewy bodies), dysfunctions of the mitochondria and lysosomes as well as neuroinflammation, which in turn leads to the death of nigrostriatal dopaminergic neurons [3]. Based on the pathophysiological knowledge that striatal dopamine depletion is the core mechanism for motor symptoms of PD, substitution with levodopa (a dopamine precursor) is the mainstay of treatment for motor symptoms. Younger patients with PD are often treated with dopamine agonists (e.g., pramipexole, ropinirole) to delay the use of levodopa due to the higher risk of developing dyskinesia, whereas older patients with PD (>70 years) usually receive levodopa-based therapy. To reduce levodopa-related side effects (e.g., nausea, vomiting, orthostatic hypotension) resulting from the conversion of levodopa to dopamine outside the central nervous system via DOPA decarboxylase, levodopa is routinely given in combination with peripheral DOPA decarboxylase inhibitors (DCI) such as carbidopa or benserazide [4,5]. Levodopa/DCI combinations are central substances in the treatment of both PD and restless legs syndrome (RLS) and no significant difference in therapeutic effects or adverse reactions has been observed between both of the abovementioned levodopa/DCI combinations (carbidopa or benserazide) [6,7].

Clinically, PD is diagnosed as part of a three-step process based on criteria defined by the International Parkinson and Movement Disorder Society, whereby the occurrence of bradykinesia and at least one additional cardinal motor feature (rigidity or rest tremor) is absolutely necessary to make the diagnosis [8,9]. Typically, the diagnosis of PD arises with the onset of these motor symptoms but their onset may have been preceded by a “prodromal phase” with typical non-motor symptoms (e.g., REM sleep behavior disorder, depression, hyposmia, anxiety) for several years [10]. Autonomic dysfunctions (e.g., orthostatic hypotension, fatigue, urinary dysfunction, etc.) affecting up to 70% of PD patients become even more prevalent during the course of PD and significantly reduce patients’ quality of life [11]. With regard to urinary dysfunction, it is hypothesized that in patients with PD, the depletion of dopaminergic neurons in the substantia nigra in parallel with the loss of ventral tegmental area mesolimbic dopaminergic fibers impairs neurogenic bladder control, leading to severe bladder dysfunctions [12,13]. It is important to note that the occurrence of lower urinary tract infections (LUTI) is one of the leading causes of acute hospitalization in patients with PD [14,15]. In practice, multiple factors such as altered urodynamics (detrusor overactivity and resulting urge incontinence, increased residual urine volume), and, especially in patients with PD-related dementia, frailty and diminished cognitive reserve increase the risk of LUTI [14,16,17]. Importantly, a recently published systematic review and meta-analysis reported a pooled prevalence of lower urinary tract symptoms (LUTS) in PD of 61% and a pooled prevalence of storage symptoms and voiding symptoms of 59% and 24%, respectively [18]. In light of this connection, several studies have analyzed the impact of dopaminergic drugs on urinary dysfunctions with conflicting results [19]. However, there is no evidence in the literature of any connection with the occurrence of LUTI when comparing the most commonly used antiparkinsonian medications. Aiming to explore the topic more thoroughly, we conducted a retrospective cohort study and included patients with an initial prescription of levodopa-based therapy including fixed-dose combinations of levodopa + benserazide and levodopa + carbidopa to analyze the incidence of LUTI and antibiotic drug prescriptions.

## 2. Materials and Methods

### 2.1. Database

This study was based on data from the Disease Analyzer database (IQVIA), which contains drug prescriptions (e.g., ATC codes, dosage scheme, date of visit, product and quantity), diagnoses (e.g., date of diagnosis, ICD codes, referrals, sick leave), and basic medical (e.g., BMI, blood parameter) and demographic data (e.g., sex, age, type of health insurance) obtained directly and in anonymous format from computer systems used in the practices of general practitioners and specialists [18]. The database covers approximately 3% of all outpatient practices in Germany. Diagnoses (in accordance with the International Classification of Diseases, 10th revision (ICD-10)), prescriptions (in accordance with the Anatomical Therapeutic Chemical (ATC) classification system), and the quality of the reported data are monitored by IQVIA. In Germany, the sampling methods used to select physicians’ practices are appropriate for obtaining a representative database of general and specialized practices. It has previously been shown that the panel of practices included in the Disease Analyzer database is representative of general and specialized practices in Germany [18]. Finally, this database has already been used in previous studies focusing on urinary tract infections [20,21].

### 2.2. Study Population

This retrospective cohort study included adult patients (≥18 years) with an initial prescription of levodopa therapy including fixed-dose combinations of levodopa + benserazide and levodopa + carbidopa in 1284 general practices in Germany between January 2010 and December 2020 (index date); patients with lower urinary tract infections (ICD-10: N39.0) or acute cystitis (ICD-10: N30.0) within 12 months prior to or on the index date were excluded. 

Patients treated with levodopa + benserazide and levodopa + carbidopa were matched to each other by propensity scores based on sex, age, and diagnoses documented within 12 months prior to or on the index date including diabetes (ICD-10: E10–E14), Parkinson’s disease (ICD-10: G20, G21), restless legs syndrome (RLS) (ICD-10: G25.81), benign prostate hyperplasia (ICD-10: N40), and urinary incontinence (ICD-10: N39.3, N39.4). Based on current literature it is well known that these diagnoses can be associated with an increased risk of LUTI. As the dose of levodopa is different in levodopa + benserazide and levodopa + carbidopa fixed-dose combinations, only patients receiving a 100 mg dose of levodopa were included (both combinations contain 100 mg doses which makes up 80% of prescriptions).

### 2.3. Study Outcomes

The main outcome of the study was the incidence of LUTI and antibiotic drug prescription within 12 months after the index date as a function of levodopa fixed-dose combination therapy. Each patient was followed up from the index date for a period of up to 12 months until the first LUTI diagnosis was documented or study therapy ended.

### 2.4. Statistical Analyses

Differences in the sample characteristics between patients treated with levodopa + benserazide and those receiving levodopa + carbidopa were tested using McNemar tests for all variables except the continuous age variable (Wilcoxon signed-rank test). The cumulative incidence of LUTI was estimated using the Kaplan–Meier method. Conditional Cox regression models were used to study the association between levodopa (100 mg) + benserazide compared to levodopa (100 mg) + carbidopa and LUTI incidence. These models were prepared separately for women, men, three age groups, and patients with Parkinson’s disease and RLS. As a sensitivity analysis, the outcome was also defined as a diagnosis of LUTI plus a prescription of antibiotic drugs (ATC: J01) within seven days following the LUTI diagnosis. Both regression models were adjusted for physicians’ practices to reflect the diagnosis behavior of treating physicians. To counteract the problem of multiple comparisons, *p*-values < 0.01 were considered statistically significant. Analyses were carried out using SAS version 9.4 (SAS Institute, Cary, CA, USA).

## 3. Results

### 3.1. Basic Characteristics of the Study Sample

**Figure 1:**
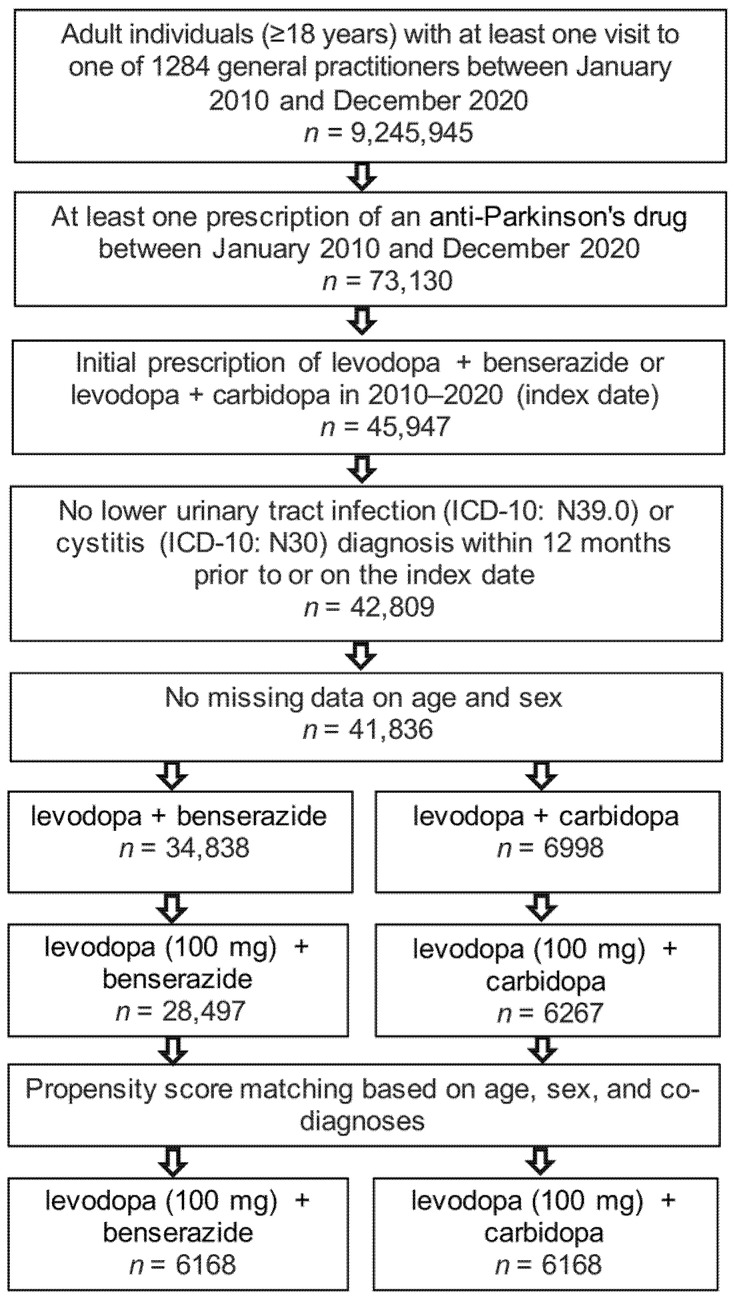
Selection of study patients.

The present study included 6168 patients receiving levodopa (100 mg) + benserazide therapy and 6168 patients with levodopa (100 mg) + carbidopa prescriptions. The selection of study patients is shown in Figure 1 and the basic characteristics of study patients are displayed in Table 1. Due to the matched pair design, all six cohorts had an identical age, sex, and comorbidity structure. The mean age was 76.1 years; 55% of patients were women, 53% had Parkinson’s disease, 23% RLS, and no diagnosis for prescription was documented for 24%.

### 3.2. Cumulative Incidence of LUTI Diagnosis

**Figure 2:**
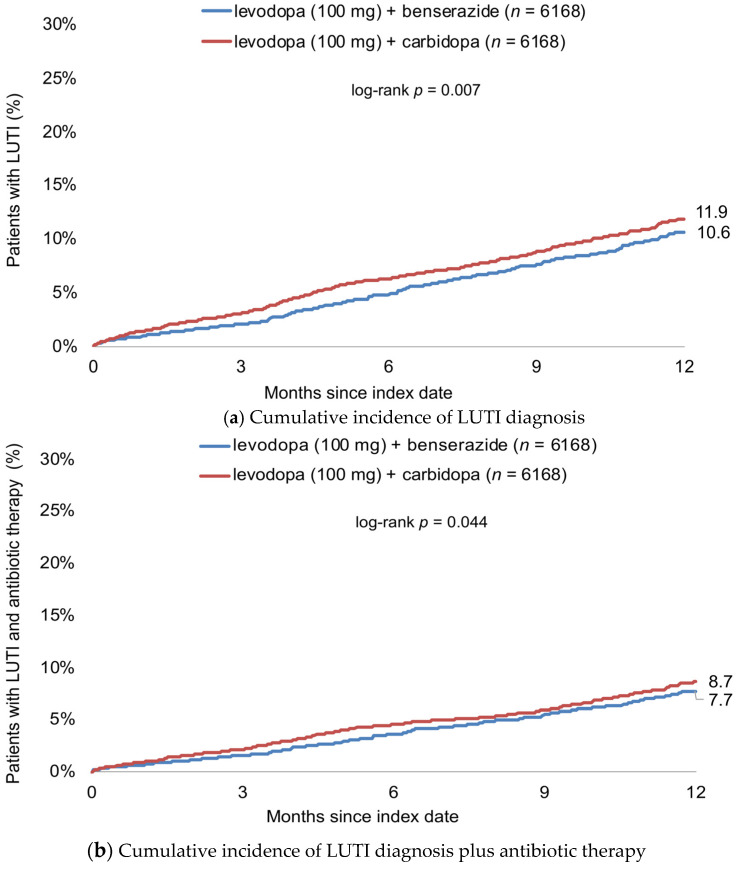
Cumulative incidence of LUTI diagnosis (**a**) and antibiotic therapy (**b**) within 12 months after start of therapy in patients treated with levodopa (100 mg) + benserazide and levodopa (100 mg) + carbidopa.

Figure 2 shows the cumulative incidence of LUTI diagnosis. Twelve months after start of therapy, this cumulative incidence was 10.6% in patients treated with levodopa (100 mg) + benserazide and 11.9% in patients receiving levodopa (100 mg) + carbidopa (Figure 2a). Antibiotic therapy was prescribed in 6.7% of patients receiving levodopa (100 mg) + benserazide and 7.7% of patients treated with levodopa (100 mg) + carbidopa (Figure 2b).

### 3.3. Association between Levodopa-Based Therapy and Incidence of LUTI

Table 2 shows the results of conditional regression analyses. Compared to levodopa (100 mg) + carbidopa, levodopa (100 mg) + benserazide therapy was significantly and negatively associated with LUTI (HR: 0.82; 95% CI: 0.71–0.95). This association was stronger in women (HR: 0.77; 95% CI: 0.65–0.92) than in men (HR: 0.93, not significant). The strongest association was observed in patients with RLS (HR: 0.65; 95% CI: 0.47–0.90). The association was significant in the age group 71–80, but not in age groups ≤70 and >80. The negative association between levodopa (100 mg) + benserazide and LUTI with antibiotic prescription was only significant in women (HR: 0.76; 95% CI: 0.62–0.93). HR was 0.64 in RLS patients, but the significance value did not reach the *p* < 0.01 value.

## 4. Discussion

Our study identifies that treatment with a combination of levodopa + benserazide was significantly and negatively associated with the incidence of LUTI (HR: 0.82; 95% CI: 0.71–0.95) compared to treatment with levodopa + carbidopa. This association was stronger in women (HR: 0.77; 95% CI: 0.65–0.92) than in men (HR: 0.93, not significant). In terms of clinical relevance, these observations have far-reaching clinical consequences, since it is known that patients with PD are per se at higher risk of developing LUTI (48.6% vs. 23.3%, *p* < 0.001) compared to non-PD controls [22]. Notably, an ascending urinary tract infection can also result in urosepsis with mortality rates ranging from 25% to 60% [23]. Based on our results, it is conceivable that PD patients with the highest susceptibility to LUTI should be treated with levodopa + benserazide rather than levodopa + carbidopa. This may include PD patients with urological dysfunctions (detrusor hyporeflexia, bladder outlet obstruction), advanced disease progression (progressive motor and non-motor dysfunction, dementia), and other more general non-modifiable LUTI risk factors (e.g., age and sex). It is even more important for clinicians to bear this information in mind when deciding on the most appropriate therapy for a PD patient, since it is known that LUTI can induce immune mediated neurological dysfunction and thereby trigger acute cerebral dysfunctions [24]. Mechanistically, bacteria mediate an immune response in patients with urinary tract infections due to the release of proinflammatory cytokines such as interleukin 6 (IL-6) and interleukin 8 (IL-8) [25]. However, it has been shown in animal models that mice with UTIs have significantly greater impairments in frontal and hippocampus-mediated behaviors than control mice without UTIs [26]. In addition, bacterial endotoxins can influence alpha-synuclein production and aggregation, thereby accelerating the death of nigrostriatal neurons. In summary, LUTI-induced systemic inflammation can also lead to neuronal PD-related neurological dysfunction [14,27,28].

Unfortunately, only a few studies published several decades ago have compared both levodopa/DCI combinations exclusively with respect to the beneficial effects on parkinsonian disability and individual symptoms. For example, Admani et al. published a randomized double-blind study where 60 patients suffering from PD were treated with levodopa + benserazide or levodopa + carbidopa. Both drug combinations effectively improved the disability scores for the parkinsonian symptoms, although the combination of levodopa + benserazide showed more improvement for all parkinsonian signs and symptoms compared to levodopa + carbidopa (however, the differences were not statistically significant) [29]. Greenacre et al. conducted a blind randomized crossover trial with a small cohort of 19 PD patients and compared the therapeutic efficacy and side effects of both levodopa/DCI combinations. In this study, the authors observed no significant difference between the treatment regimens with regard to the beneficial effects on parkinsonian symptoms or the occurrence of adverse effects [7]. Another group investigated the pharmacokinetics of both levodopa/DCI combinations and found some interesting differences. For example, the administration of levodopa/benserazide provided a higher levodopa maximum observed plasma concentration (C_max_) and larger under the plasma concentration-time curve from time 0 to 3 h (AUC_0–3h_) than levodopa/carbidopa. Based on these findings, the authors concluded that levodopa/benserazide might be a better therapeutic option for patients with more severe adverse effects or inadequate levodopa efficacy. Despite showing significant differences in levodopa pharmacokinetics when combining levodopa with different DCIs, this study did not correlate pharmacokinetic differences with the clinical outcomes of PD patients (e.g., impact on parkinsonian symptoms, occurrence of side effects) [30]. However, literature explaining differences between levodopa + benserazide and levodopa + carbidopa therapies with a view on LUTI incidence and urinary dysfunctions is sparse. Especially, there are no randomized clinical trials or meta-analyses available comparing the levodopa/DCI combinations and thereby focusing on the occurrence of urinary tract infections. One study published by Zhu et al. shows that carbidopa potently inhibits T cell responses and T cell-mediated autoimmunity in two different animal models, demonstrating a immunosuppressive activity of this DCI. The authors concluded that PD patients treated with carbidopa may show immune suppression and thus will be at enhanced risk of infection. Finally, this could be an explanation for the superiority of levodopa + benserazide [31]. Notably, several management strategies regarding the prevention and treatment of LUTI in patients with PD have been already published. These include preventive recommendations such as vaginal estrogen supplementation, use of cranberry products, and taking vitamins and other supplements, as well as interventions such as clean catheterization and continuous low-dose antibiotic prophylaxis [32]. Given that LUTI is a leading cause of hospitalizations, morbidity, and mortality in patients with PD, further research is necessary to optimize the pharmacological treatment of PD, taking into account the urological dysfunctions and infections that often accompany it [14,22].

Due to the design of our study, patients diagnosed with RLS receiving both levodopa/DCI combinations were also included. The strongest negative association between levodopa + benserazide compared to levodopa + carbidopa and LUTI was observed for these patients (HR: 0.65; 95% CI: 0.47–0.90). Using the SCOPA-AUT scale, which determines the presence and frequency of autonomic symptoms, a recent study showed that RLS patients have significantly more urological dysfunctions than patients in the control group [33,34]. However, there is a lack of literature comparing and analyzing various dopaminergic treatment options in RLS patients with respect to severe autonomic impairments (e.g., urologic dysfunctions) and their consequences, such as LUTI.

Another interesting finding of our study refers to specific sex-related factors: we observed that the negative association between levodopa + benserazide and LUTI (compared to levodopa + carbidopa) was stronger in women (HR: 0.77; 95% CI: 0.65–0.92) than in men (HR: 0.93, not significant). Furthermore, the negative association between levodopa + benserazide and LUTI with antibiotic prescription was only significant in women (HR: 0.76; 95% CI: 0.62–0.93). Currently, PD treatment may differ based on patients’ symptoms, age etc., but the choice between different drug treatments is still not sex-oriented. It is important to note that levodopa is still known to have a significantly higher bioavailability and lower clearance level in women [35,36]. Interestingly, these sex-related differences were also shown in a multicentric study on levodopa-naive PD patients receiving levodopa/benserazide for the first time. The authors of this study found that female sex and body mass index significantly predicted area under the curve (AUC) and maximum concentration (C_max_) [37,38]. Based on these data, it is conceivable that sex differences may result in different clinical responses to PD treatment and thereby influence clinical features of PD, accounting for increased LUTI risk in a different way.

## 5. Strengths and Limitations

Our retrospective cohort study has several strengths. The German Disease Analyzer (DA) is a large German outpatient database containing data from 2898 practices with about 7.8 million patients in Germany during the study period. The representativeness of the diagnoses it contains has already been validated [39,40]. Furthermore, the DA database provides continuously updated encrypted data generated directly from practice computers based on patient data (diagnoses, demographic data, prescriptions, specific measurements such as blood pressure, BMI, blood glucose, and serum lipids) via interfaces. To further increase the quality of our study, we also included several co-diagnoses within the propensity score matching that increase the risk of urinary retention and LUTI per se, such as benign prostatic hyperplasia or urinary incontinency.

However, the DA does not contain information on external confounding factors (alcohol and tobacco consumption, socioeconomic status, etc.). Moreover, the lack of hospital data and information on mortality should also be considered. Furthermore, diagnoses are based solely on ICD-10 codes documented by general practitioners and do not include diagnoses by gynecologists, neurologists, or urologists, which could influence the quality of the diagnoses documented. In addition, there is a lack of detailed information (e.g., types of parkinsonism, frequency of autonomic dysfunctions, treatment duration, urodynamic data), and physicians’ motives (indications) for prescribing specific anti-Parkinson’s medications were therefore not available for this study. 

## 6. Conclusions

LUTI is one of the leading causes of hospitalization and mortality in patients with PD due to the increased risk of developing urosepsis. We identified a levodopa/DCI combination (levodopa + benserazide) as being significantly and negatively associated with LUTI compared to levodopa + carbidopa, while the antiparkinsonian efficacy of both levodopa/DCI combinations is the same. This result has far-reaching clinical consequences, especially for PD patients with the highest LUTI risk (urological dysfunction, PD-related dementia etc.) since a levodopa + benserazide combination should be preferred for such patients.

## Figures and Tables

**Table 1:** Basic characteristics of the study sample after propensity score matching.

Variable	Proportion among Patients Treated with Levodopa (100 mg) + Benserazide (%)	Proportion among Patients Treated with Levodopa (100 mg) + Carbidopa (%)	*p*-Value
***n***	6168	6168	
**Age (Mean, SD)**	76.1 (11.4)	76.1 (11.4)	0.925
**Age ≤ 70**	23.7	23.9	0.982
**Age 71–80**	34.7	35.0	
**Age > 80**	41.6	41.2	
**Female**	54.4	54.2	0.828
**Male**	45.6	45.8	
**Diabetes**	28.2	28.2	0.968
**Parkinson’s disease**	52.6	52.6	0.971
**RLS**	23.4	23.3	0.932
**Benign prostate hyperplasia**	11.5	11.6	0.822
**Urinary incontinency**	8.1	8.4	0.577

Proportions of patients are given in % unless otherwise indicated. SD: standard deviation.

**Table 2:** Association between levodopa combination therapy and LUTI diagnosis as well as antibiotic prescriptions in patients followed in general practices in Germany (Cox regression models).

Cohort	LUTI		LUTI + Antibiotic Therapy	
	HR (95% CI) for Levodopa (100 mg) + Benserazide Compared to Levodopa (100 MG) + Carbidopa	*p*-Value	HR (95% CI) for Levodopa (100 mg) + Benserazide Compared to Levodopa (100 mg) + Carbidopa	*p*-Value
**Total**	0.82 (0.71–0.95)	0.007	0.84 (0.71–1.00)	0.045
**Age ≤ 70**	0.82 (0.57–1.16)	0.261	0.92 (0.60–1.40)	0.690
**Age 71–80**	0.70 (0.54–0.90)	0.006	0.79 (0.59–1.06)	0.118
**Age > 80**	0.90 (0.74–1.10)	0.287	0.84 (0.67–1.07)	0.156
**Women**	0.77 (0.65–0.92)	0.004	0.76 (0.62–0.93)	0.009
**Men**	0.93 (0.73–1.18)	0.531	1.04 (0.78–1.40)	0.792
**Parkinson’s disease**	0.82 (0.67–1.01)	0.063	0.85 (0.67–1.08)	0.179
**RLS**	0.65 (0.47–0.90)	0.008	0.64 (0.44–0.93)	0.019

Proportions of patients are given in % unless otherwise indicated. SD: standard deviation.

## Data Availability

Anonymized raw data are available upon reasonable request.
